# New Frontiers in Locally Advanced Cervical Cancer Treatment

**DOI:** 10.3390/jcm13154458

**Published:** 2024-07-30

**Authors:** Roberta Massobrio, Lavinia Bianco, Beatrice Campigotto, Daniela Attianese, Elisa Maisto, Maria Pascotto, Maria Grazia Ruo Redda, Annamaria Ferrero

**Affiliations:** 1 Academic Division of Gynecology and Obstetrics, University of Turin, Mauriziano Umberto I Hospital, 10128 Turin, Italy; roberta.massobrio@edu.unito.it (R.M.); beatrice.campigotto@unito.it (B.C.); d.attianese2807@gmail.com (D.A.); elisa.maisto@unito.it (E.M.); maria.pascotto@unito.it (M.P.); 2 Department of Radiation Oncology, University of Turin, Mauriziano Umberto I Hospital, 10128 Turin, Italy; lvbianco@mauriziano.it (L.B.); mariagrazia.ruoredda@unito.it (M.G.R.R.); 3 Department of Surgical Sciences, University of Turin, 10124 Turin, Italy

**Keywords:** locally advanced cervical cancer, chemoradiation therapy, neoadjuvant chemotherapy, adjuvant chemotherapy, immunotherapy, immune checkpoint inhibitors

## Abstract

Despite the introduction of targeted vaccines and screening protocols, locally advanced cervical cancer represents a median proportion of 37% among all cervical carcinomas. Compared to early stages, it presents significantly lower cure rates, with a 5-year disease-free survival rate of 68% and a 5-year overall survival rate of 74%. According to current guidelines, definitive radiotherapy with concomitant chemotherapy represents the gold standard for locally advanced cervical cancer treatment. However, a significant number of patients relapse and die from metastatic disease. The aim of this narrative review is to examine the recent advancements in treating locally advanced cervical cancer, exploring new frontiers in therapeutic approaches. The PubMed database and clinical trial registries were searched to identify relevant articles published on locally advanced cervical cancer treatment up to March 2024, mainly focusing on papers published in the last decade. Abstracts presented at major international congresses that bring relevant evidence were included. Progress achieved in refining radiotherapy techniques, recent evidence regarding neoadjuvant treatment preceding surgery or concurrent chemoradiotherapy, and key findings concerning adjuvant treatment are thoroughly explored. Furthermore, a comprehensive review of prominent phase II and phase III trials examining the integration of immune checkpoint inhibitors is conducted, analyzing the various contexts in which they are applied. In light of the new evidence that has emerged in recent years and is discussed in this article, the appropriate selection of the most suitable therapeutic approach for each patient remains a complex but crucial issue.

## 1. Introduction

Cervical cancer is the fourth most frequently diagnosed cancer and it represents the fourth leading cause of cancer death in women, with 661,021 estimated new cases and 348,189 deaths worldwide in 2022 [1].

In high-income countries, the widespread introduction of screening programs and the implementation of human papillomavirus vaccines have led to a considerable decrease in incidence and earlier diagnosis. However, many patients still present advanced disease at the onset, especially in low-resource countries. Locally advanced cervical cancer (LACC), defined as stages IB3–IVA of the disease according to the FIGO 2018 classification [2], represents a median proportion of 37% of all cervical cancer cases worldwide, reaching approximately 90% in countries with limited resources and lower socioeconomic conditions [3].

Compared to early stages, locally advanced cervical cancer has significantly lower cure rates, with a 5-year disease-free survival rate of 68% and a 5-year overall survival rate of 74%; prognostic factors for poorer outcomes include higher disease stage and lymph node involvement [4,5].

While surgery is the principal treatment for early-stage disease, providing excellent outcomes, for patients with locally advanced cervical cancer, concomitant cisplatin-based chemoradiation (CCRT) followed by intrauterine brachytherapy (BT) is recommended by the ESGO/ESTRO/ESP Guidelines [6]. This integrated approach, adding concurrent chemotherapy to radiotherapy, has been demonstrated to enhance the therapeutic efficacy and overall survival rates [7,8,9], and the addition of brachytherapy represents a crucial element for optimal local control [10]. However, a considerable number of patients experience recurrence and die from metastatic disease, making advanced cervical cancer treatment an area with unmet needs.

In this scenario, many recent advances, such as the refinement of radiotherapy techniques and the introduction of novel treatment options, including immunotherapy agents, are contributing to bringing new perspectives to the management of this pathology.

The aim of this narrative review is to examine the recent advancements in treating locally advanced cervical cancer, exploring new frontiers in therapeutic approaches.

## 2. Materials and Methods

For the present narrative review, the PubMed database was searched to identify relevant articles on locally advanced cervical cancer treatment published up to March 2024, mainly focusing on papers published in the last decade. The following terms were used: (“chemoradiation” OR “chemoradiotherapy”) AND “locally-advanced cervical cancer”, “brachytherapy” AND “locally-advanced cervical cancer”, “neoadjuvant” AND “locally-advanced cervical cancer”, “adjuvant” AND “locally-advanced cervical cancer”, “locally-advanced cervical cancer cervical cancer” AND (“immunotherapy” OR “immune checkpoint inhibitor”). Additional publications were identified via a review of all reference lists within the identified publications. Only publications written in English were included. Additionally, clinical trial registries were searched, and abstracts presented at major international congresses that bring relevant evidence were included in the analysis. An initial selection was made by title and abstract; subsequently, original papers were reviewed and only prospective studies with the greatest clinical impact were selected. All findings were combined into a narrative description divided into different paragraphs corresponding to the topics we aimed to investigate.

## 3. Results

### 3.1. Definitive Chemoradiation and Brachytherapy

The standard treatment of locally advanced cervical cancer patients is external beam radiotherapy (EBRT) and concomitant chemotherapy, followed by brachytherapy. The trials on CCRT and brachytherapy examined in this review are summarized in Table 1.

In radiation therapy, identifying the appropriate extension of the radiotherapy field is crucial. In locally advanced cervical cancer, this process is guided by assessing pelvic and para-aortic node involvement and the presence of extrapelvic disease through Positron Emission Tomography-Computed Tomography (PET-CT) [6]. Unfortunately, even PET-CT has a moderate false-negative rate, especially with regards to para-aortic nodes [11,12,13]; therefore, surgical staging through a minimally invasive lymph node dissection can be an option [14].

The international multicenter phase III Uterus-11 trial randomized 240 patients with FIGO 2009 stages IIB-IVA cervical cancer to surgical or clinical staging followed by platinum-based chemoradiation. For the experimental arm, a pelvic and paraaortic lymphadenectomy was performed and the selected approach was laparoscopic in 96.6% of patients. Clinical staging was conducted through abdominal CT and/or abdominal magnetic resonance + chest imaging. After a median follow-up of 90 months, no difference in disease-free survival (DFS) was observed between the two groups (*p* = 0.084); however, surgical staging was associated with significantly improved DFS in patients with FIGO stage IIB disease (HR 0.51, *p* = 0.011). Moreover, in a post hoc analysis, surgical staging was associated with better cancer-specific survival (HR 0.61, *p* = 0.020) [15].

Last year the PAROLA Study group launched the multicenter, randomized, phase III PAROLA trial (NCT05581121) [16], recruiting PET-CT FIGO stage IIIC1 patients. The primary objective of the study is to evaluate the benefit in terms of DFS of chemoradiation with tailored EBRT field based on surgical staging and pathological examination of para-aortic nodes compared with patients clinically staged with PET-CT. Secondary endpoints include overall survival, cancer specific-survival, metastasis-free survival, surgical and radiation morbidities, quality of life, and cost analyses. Accrual completion is estimated for Q2 2027.

Intensity-Modulated Radiation Therapy (IMRT) has emerged as a promising method that uses small beamlets that can vary in intensity, leading to a better conformation to three-dimensional target volumes, while reducing dose to organs at risk (OARs) if compared conventional radiotherapy [17]. The potential of IMRT to reduce the dose to bone marrow during pelvic RT has acquired significant interest, aiming to mitigate the hematological toxicity associated with pelvic radiotherapy in patients undergoing concomitant chemotherapy. The simultaneous integrated boost (SIB) technique can be used to increase doses to the primary tumor and positive lymph nodes relative to elective nodal regions. This approach has been well tolerated and has achieved good local control [18].

The international multicenter single-arm phase II INTERTECC trial (NCT01554397) [19] treated 83 patients with stage IB-IVA cervical cancer with IMRT and concurrent platinum-based chemotherapy. The primary endpoint was the occurrence of either acute grade 3 neutropenia or gastrointestinal toxicity in the first month after the completion of chemoradiotherapy. The incidence of any primary event was 26.5%, which was significantly lower than the 40% incidence expected from historical data (*p* = 0.02). The phase III trial randomized patients to PET-based bone marrow-sparing (BMS) Image-Guided (IG)-IMRT vs. IMRT, with a primary endpoint of progression-free survival (PFS), but the study was closed early for futility. Phase III patients toxicities were analyzed separately and in combination with phase II patients and PET-based BMS-IG-IMRT presented a significantly lower incidence of acute grade ≥3 neutropenia for randomized patients (*p* = 0.048) and in the combined cohort (*p* = 0.01) [20].

A recent single center randomized trial by Huang et al. [21] enrolled 164 patients with stage Ib2–IIIb cervical cancer who were treated with concurrent chemoradiation, in order to evaluate the efficacy of pelvic bone marrow sparing (PBMS) IMRT in reducing G2 or higher hematological toxicity. The incidence of G2 hematological toxicity was significantly reduced in the PBMS arm (*p* = 0.02). They even analyzed dosimetric parameters of different subsites of bone marrow and the radiation dose to lumbosacral spine had the strongest association with hematological toxicity.

A further evolution of IMRT technique is represented by Volumetric Modulated Arc Therapy (VMAT), in which the radiation dose is continuously delivered, as the gantry of the linear accelerator (LINAC) rotates around the patient through single or multiple arcs. VMAT offers a highly precise dose distribution to the tumor by dynamically modulating the intensity of the radiation beam, the dose rate, and the gantry rotation speed. This results in faster treatment times and reduced monitor units compared to conventional IMRT [22].

Brachytherapy has been used for the treatment of cervical cancer since 1903 and has been associated with improved pelvic control and overall survival, establishing the technique as an essential component of definitive treatment for cervical cancer [6,17]. Brachytherapy performed at the end of EBRT allows for dose escalation to the primary site of disease with a rapid dose fall-off in organs at risk near the tumor.

Numerous technological advancements have emerged in the field of brachytherapy: the most important is image-guided adaptive brachytherapy (IGABT), which consists of implementation of magnetic resonance imaging (MRI) before and during brachytherapy administration, being able to realize an adaptive, three-dimensional planning. Indeed, IGABT incorporates tumor and OAR anatomy and positioning into treatment planning and enables a paradigm shift from prescribing the dose to an arbitrary point to the actual primary target while monitoring and appropriately sparing adjacent normal organs [17].

In 2021, Potter et al. published the results of the EMBRACE I study [4], a prospective observational study with an accrual of 1416 patients, designed to assess outcomes from application of MRI-based IGBT in a multicenter, international population according to standards developed by the Gynaecological Groupe Europeen de Curietherapie and the European Society for Radiotherapy and Oncology (Gyn GEC-ESTRO). At a median follow-up of 51 months, the overall 5-year local control was 92%; also, patients with stage IVA disease had local control of 76% at 5 years. The 5-year disease-free survival (DFS) in this cohort was 68%, and 5-year OS was 74%. The GEC-ESTRO-GYN network also carried out a retrospective collection of data (called retroEMBRACE) on 852 patients treated with IGABT before the start of EMBRACE I, highlighting the excellent outcomes associated with this technique [23].

In 2016, the EMBRACE II study [24] started as a prospective interventional study with specific treatment interventions based on the outcome results of the retroEMBRACE and EMBRACE I studies. The aim is to validate the findings from previous EMBRACE studies and to enhance survival rates, by improving local, nodal, and systemic control, while reducing morbidity and improving quality of life. Great attention is paid to EBRT fields, doses, vaginal constraints, and technical details regarding brachytherapy. Study completion is estimated in April 2031.

Regarding radiotherapy fractionation, in the last twenty years, only a few studies have explored the use of hypofractionation in cervical cancer radiotherapy. The results seemed promising, but they were mostly Phase I-II trials or retrospective reports with significant heterogeneity between them. Furthermore, older techniques were used, so it has not been possible to draw definitive conclusions yet. In any case, there are some interesting ongoing trials aiming to investigate the role of hypofractionation in cervical cancer patients [25].

In Canada, an important multicentric phase II randomized trial, the Hypofractionated External Beam Radiotherapy for Intact Cervical Cancer (HEROICC) study [26], is comparing hypofractionated RT versus standard treatment. The trial is recruiting low-risk LACC patients with limited nodal involvement. FIGO 2018 stages IA to IIB patients are included if they are not candidates for surgical treatment. In addition, patients with FIGO stage IIIC1 disease and no common iliac nodal disease, pelvic lymph nodes (<3 cm in largest dimension), and less than three pathologic nodes are eligible. In the experimental arm, RT is given with a total dose of 40 Gy in 15 fractions with concurrent chemotherapy followed by brachytherapy. Positive nodes are boosted to a dose of 46–48 Gy with the SIB technique. Standard treatment gives a VMAT dose of 45 Gy in 25 fractions and 55–57.5 Gy to involved nodes. The primary endpoint is the feasibility of patient enrollment in the Canadian healthcare system; the secondary outcomes include tumor downstaging on magnetic resonance (MR) imaging at the time of brachytherapy, bowel, urinary, and sexual quality of life, and survival endpoints such as locoregional progression-free survival and metastasis-free survival. Study completion is estimated in December 2028.

A second trial is being conducted at Tehran University, with the aim of assessing if hypofractionated radiotherapy is non-inferior to standard treatment in terms of clinical response and toxicity. The enrolled patients are stage IB-IIIC and in the experimental arm, they are submitted to 40 Gy EBRT in 15 fractions in addition to chemotherapy followed by brachitherapy, similar to the above-mentioned Canadian study. Study completion is estimated in March 2028 [27].

The phase II randomized Thai HYPOCx-iRex trial is comparing hypofractionated RT with a dose of 44 Gy in 20 fractions to the pelvis and 53 Gy in 20 fractions to positive lymph nodes with concomitant chemotherapy to the standard treatment (45 Gy/25 fr and 55 Gy to lymph nodes plus chemotherapy). The target contour and planning dose of EBRT and IGABT were adapted from the EMBRACE II study protocol. Preliminary results were reported at ASTRO 2023: among twenty patients with 8 months’ follow-up, there was one with grade 3 early toxicity in the experimental arm but no grade 3 late GI toxicity; furthermore, oncological outcomes and patterns of failure were similar [25].

Stereotactic body radiotherapy (SBRT) is another type of EBRT whereby high doses of radiation per fraction are delivered with high precision to a target in one or a few fractions. In recent years, SBRT has been evaluated as a conformal RT boost alternative to brachytherapy, particularly in patients unable to undergo to BT due to unfavorable anatomy or medical comorbidities [17].

A recent single-arm phase II trial [28] of SBRT boost as an alternative for intracavitary/interstitial BT boost for LACC was conducted and published in 2020, but the trial closed prematurely after 15 patients were enrolled because of toxicity concerns. Indeed, 2-year cumulative grade ≥ 3 toxicity was 26.7%, with many cases of rectal ulcers/fistulas. Two of the grade 3 patients died of complications from fistulas, resulting in a grade 5 toxicity rate of 13%. Moreover, the efficacy of SBRT was lower than expected, showing 2-year local control, progression-free survival, and overall survival rates of 70.1%, 46.7%, and 53.3%, respectively. Therefore, this trial confirmed the importance of brachytherapy, which, as stated in ESGO guidelines, “is an essential component of definitive radiotherapy and should not be replaced with an external boost (photon or proton). If BT is not available, patients should be referred to a center where this can be done” [6].

**Table 1:** CCRT and brachytherapy in locally advanced cervical cancer.

Clinical Trial Registry Identifier	Title	Phase	Patients	Intervention	Primary Endpoint (s)	Outcome (s)
	Surgical versus clinical staging prior to primary chemoradiation in patients with cervical cancer FIGO stages IIB–IVA: oncologic results of a prospective randomized international multicenter (Uterus-11) intergroup study [15]	Phase III RCT	240	Surgical versus clinical staging	DFS	No difference in DFS; significant benefit in DFS for FIGO stage IIB
NCT05581121	PARa-aOrtic LymphAdenectomy in locally advanced cervical cancer (PAROLA trial): a GINECO, ENGOT, and GCIG study [16]	Phase III RCT	510 (estimated)	Surgical staging versus clinical staging	DFS	Accrual completion estimated in Q2 2027
NCT01554397	Bone Marrow-sparing IMRT With Concurrent Cisplatin For Stage IB-IVA Cervical Cancer: An International Multicenter Phase II Clinical Trial (INTERTECC-2) [19]	Phase II (single-arm)	83	IG-IMRT versus IMRT and concurrent CT	G3 neutropenia or clinically significant acute gastrointestinal toxicity	Lower hematologic toxicity (19.3%) and GI toxicity (12.0%)
ChiCTR1800015069	Pelvic bone marrow-sparing IMRT reduces the incidence of the hematologic toxicity of patients with cervical cancer receiving concurrent chemoradiotherapy: a single-center prospective randomized controlled trial [21]	RCT (single-center)	164	Bone marrow-sparing IMRT versus IMRT	≥G2 hematological toxicity	Lower ≥ G2 hematological toxicity in PBMS IMRT (50% vs. 69.5%, *p* = 0.02)
NCT00920920	An International Study on Magnetic Resonance Imaging (MRI)-Guided Brachytherapy in Locally Advanced Cervical Cancer (EMBRACE I) [4]	Multicenter prospective observational study	1416	EBRT + 3D-(MRI)IGBT	5 y local control; 5 y morbidity	5 y local control 92%; 5 y morbidity: 6.8% genitourinary, 5.8% gastrointestinal, 5.7% vaginal, 3.2% fistulae
NCT03617133	Image-Guided IMRT, Radiochemotherapy, and MRI-based IGABT in Locally Advanced Cervical Cancer (EMBRACE II) [24]	Interventional prospective study	1000 (estimated)	EBRT (IMRT) + 3D MRI-guided brachytherapy with increased use of combined IC/IS brachytherapy	Local control; Nodal control; systemic control; OS; overall morbidity; QoL	Study completion estimated in April 2031
NCT04583254	Hypofractionated External-beam RadiOtherapy for Intact Cervical Cancer (HEROICC-Trial): A Feasibility Study [26]	Phase II	48 (estimated)	Hypofractionated EBRT (40 Gy/15 fr) + Brachytherapy	Feasibility in Canadian healthcare system	Study completion estimated in December 2028
NCT04831437	Clinical Response and Toxicity of Hypo-fractionated Chemoradiotherapy in Cervix Cancer [27]	Phase II	60 (estimated)	Hypofractionated EBRT (40 Gy/15 fr) + CT + BT	Early toxicity; Early response	Study completion estimated in March 2028
TCTR20210812003	HYPOCx-iRex (TCTR20210812003) A Phase II RCT: 44 Gy/20 F vs. 45 Gy/25 F CCRT in Cervical Cancer [25]	Phase II		Hypofractionated EBRT (44 Gy/20 fr) + CT + BT	Genitourinary and gastrointestinal toxicity; OS; DFS	Interim analysis: no difference
NCT02045433	A Phase II Trial of Stereotactic Ablative Radiation Therapy as a Boost for Locally Advanced Cervical Cancer [28].	Phase II	21	EBRT + SBRT bost (DFT 28 Gy/4 fr)	Local control	2 y local control: 70.1%

### 3.2. Neoadjuvant Chemotherapy

In recent decades, multiple trials have investigated the potential benefits of integrating neoadjuvant chemotherapy (NACHT) in locally advanced cervical cancer treatment.

A meta-analysis by Nguyen et al. [29] including 3632 patients failed to demonstrate an OS benefit of cisplatin-based neoadjuvant chemotherapy followed by local treatment compared with local treatment alone. On the other hand, dose-dense cisplatin at over 72.5 mg/m^2^/3 weeks was significantly associated with OS (*p* < 0.05) and the survival benefit was even greater when cisplatin was administered at a dose over 105 mg/m^2^/3 weeks. An important limitation of this meta-analysis was the heterogeneity of local treatments administered after neoadjuvant chemotherapy across the studies, with CCRT being used in only one study.

In this section, we present the most pertinent recently published studies (Table 2). Regarding randomized clinical trials, we focused on studies comparing neoadjuvant chemotherapy followed by surgery or concurrent chemoradiotherapy (CCRT) with standard CCRT treatment.

#### 3.2.1. Neoadjuvant Chemotherapy Followed by Chemoradiation

A Brazilian phase II prospective, non-randomized trial (NCT02309658) [30] evaluated NACHT with cisplatin and gemcitabine before CCRT in 50 patients with LACC (FIGO 1994 stage IB2–IVa). Response rate and toxicity were the primary endpoints; PFS and OS were the secondary endpoints. In this study, NACHT in locally advanced cervical cancer patients failed to demonstrate a meaningful improvement in response rate, showing an ORR of 81%. Three-year PFS and OS were 53.9% and 71.3%, respectively. Hematological and gastrointestinal toxicity were the most common. Grade 3/4 toxicity was 20% during NACHT and 44% during CCRT. Late adverse events were present in 20% of patients.

The CIRCE randomized phase II trial (NCT01973101) [31] evaluated the efficacy and safety of neoadjuvant chemotherapy with cisplatin and gemcitabine followed by CCRT. 107 patients with FIGO 2009 stages IIB to IVA locally advanced cervical cancer were randomly assigned to either NACHT followed by standard CCRT or standard CCRT alone. The neoadjuvant treatment regimen consisted in three cycles of NACHT with cisplatin 50 mg/m^2^ on day 1 and gemcitabine 1000 mg/m^2^ on day 1 and day 8 every 3 weeks for three cycles, followed by CCRT, which started 3 to 4 weeks after the last cycle of NACHT. The primary endpoint was 3-year PFS. The secondary endpoints were response rate, 3-year locoregional control, 3-year OS, safety, and quality of life. The addition of neoadjuvant chemotherapy was associated with inferior 3-year PFS rates (40.9% versus 60.4% in the CCRT arm, HR 1.84) and a lower 3-year OS rate (60.7% versus 86.8%, HR 2.79) After treatment completion, complete response rates were 56.3% in the NACHT arm and 80.3% in the CCRT arm (*p* = 0.008). Toxicities were similar in both arms; however, hypomagnesemia and neuropathy were more frequent in the NACHT group. Although NACHT was well tolerated overall, more patients in the NACHT arm discontinued concurrent chemotherapy (20% versus 5.8%).

A recently published single-center randomized trial (ChiCTR1900023257) [32] compared the efficacy of neoadjuvant chemotherapy +CCRT versus CCRT in 160 locally advanced cervical cancer patients with a tumor diameter greater than 4 cm. The regimen of the neoadjuvant chemotherapy was paclitaxel 135–175 mg/m^2^ and cisplatin at 60–80 mg/m^2^, with a course of 21 days per cycle for two consecutive cycles, followed by CCRT two weeks after the completion of chemotherapy. The primary endpoints were complete response rate and safety, and the secondary endpoints were the 1- and 2-year OS and PFS. The complete response rate in the NACHT + CCRT group was significantly higher than in the CCRT group (87.7% vs. 67.6%, *p* = 0.000). In the NACHT + CCRT group, the 1- and 2-year OS rates were significantly higher than those in the CCRT group (96% vs. 89% and 89% vs. 79%, *p* = 0.017). Moreover, the rate of recurrences and distant metastases was significantly lower with the addiction of neoadjuvant chemotherapy.

Based on the promising results obtained from the single-arm phase II CxII trial [33], the phase III INTERLACE randomized trial (NCT01566240) [34] was launched, in order to investigate whether dose-dense weekly chemotherapy before standard CCRT improves survival outcomes compared with CCRT alone. The study involved 500 patients with FIGO 2008 stages IB1/2 to IVA locally advanced cervical cancer. A total of 77% of patients had stage II disease and more than half (58%) were lymph node-negative. Patients were randomized to receive either standard CCRT or induction chemotherapy (IC) with six cycles of weekly carboplatin AUC2 and paclitaxel 80 mg/m^2^ followed by CCRT. The median interval between induction chemotherapy and CCRT was 7 days. This study demonstrated a significant benefit in terms of PFS and OS with the implementation of induction chemotherapy before CCRT: the 5-year PFS rate was 73% with IC plus CCRT and 64% with CCRT alone (HR 0.65; *p* = 0.013); 5-year OS was 80% and 72%, respectively (HR 0.61. *p* = 0.04). Grade ≥ 3 adverse events were seen in 59% of patients receiving induction chemotherapy plus CCRT and in 48% of patients receiving CCRT alone.

#### 3.2.2. Neoadjuvant Chemotherapy Followed by Radical Surgery

Historical data from a meta-analysis by Tierney et al. [35] revealed a benefit of NACHT followed by surgery compared to radiotherapy alone, with a 35% decrease in the risk of death (HR 0.65, *p* = 0.0004) and an improvement of 14% in the 5-year OS rate. However, radiotherapy without concomitant cisplatin is obsolete, as are the chemotherapy regimens administered in the studies included in this meta-analysis.

More recently, two randomized phase III trials investigated the role of neoadjuvant chemotherapy before surgery compared with concomitant chemoradiation.

A single-center trial (NCT00193739) [36] included 635 patients with 1994 FIGO stage IB2, IIA, or IIB locally advanced cervical cancer and randomized them to receive either neoadjuvant chemotherapy followed by radical hysterectomy or standard CCRT. The chemotherapy regimen selected was three cycles of paclitaxel and carboplatin every 3 weeks. The primary endpoint, 5-year DFS, was significantly lower in the NACHT arm than in the CCRT arm (69.3% versus 76.7%; HR = 1.38), while the 5-year OS rates were similar (75.4% versus 74.7%, HR = 1.025). Although rectal, bladder, and vaginal toxicity at 90 days after treatment were significantly lower in the NACHT arm, 24 months after treatment, there was no difference in rectal and bladder toxicities between the two groups; only vaginal toxicity continued to be lower in the NACHT–surgery arm.

The efficacy and safety of neoadjuvant chemotherapy and surgery in comparison to CCRT in FIGO 1994 stage IB2-IIB cervical cancer were explored by the multicenter randomized phase III trial EORTC-55994 (NCT00039338) [37]. A total of 626 patients were randomized to neoadjuvant chemotherapy followed by surgery (NACHT-S) or to standard CCRT. The NACHT-S arm received platinum-based chemotherapy at a minimum cumulative cisplatin dose of 225 mg/m^2^; surgery was performed within six weeks following the completion of NACHT, involving a Piver III-V radical hysterectomy with pelvic lymphadenectomy, optionally including para-aortic nodes. The primary endpoint was 5-year overall survival rate. The secondary endpoints were PFS, OS, toxicity, and health-related quality of life. Protocol treatment was completed in 71% patients in the NACHT-S and 82% in the CCRT arms. Treatment interruption due to toxicity occurred in 9.6% of patients in the NACHT-S arm and 7.4% in the CCRT arm. A total of 48% of patients assigned to NACHT-S underwent adjuvant radiotherapy and 8% of patients assigned to CCRT required additional surgery. The study failed to demonstrate the superiority of neoadjuvant chemotherapy followed by surgery compared with CCRT: 5-year OS was 72% for the NACHT-S arm and 76% for the CCRT arm, and the 5-year PFS rates were 57% vs. 66% in the NACHT-S and CCRT groups, respectively.

**Table 2:** Neoadjuvant chemotherapy in locally advanced cervical cancer.

Clinical Trial Registry Identifier	Title	Phase	Patients	Drugs and Schedule	Primary Endpoint (s)	Outcome (s)
NCT02309658	Neoadjuvant Chemotherapy in Locally Advanced Cervical Cancer Patients [30]	II	50	gemcitabine 1000 mg/m^2^ + cisplatin 35 mg/m^2^ on day 1 and 8 for two cycles → CCRT	Response rate and toxicity	ORR 81%; G 3/4 toxicity: 20% during NACHT; 44% during CCRT
NCT01973101	Neoadjuvant Chemotherapy with Cisplatin and Gemcitabine Followed by Chemoradiation versus Chemoradiation for Locally Advanced Cervical Cancer: A Randomized Phase II Trial (CIRCE) [31]	II	107	cisplatin 50 mg/m^2^ on day 1 and gemcitabine 1000 mg/m^2^ on day 1 and 8 q3w for 3 cycles → CCRT 3 to 4 weeks after the completion of chemotherapy.	PFS	3-year PFS 40.9% (vs. 60.4% in CCRT arm, HR 1.84)
ChiCTR1900023257	Improving the efficacy and safety of concurrent chemoradiotherapy by neoadjuvant chemotherapy: a randomized controlled study of locally advanced cervical cancer with a large tumor [32]	IV	160	paclitaxel 135–175 mg/m^2^ + cisplatin at 60–80 mg/m^2^ q3w for 2 cycles → CCRT 2 weeks after the completion of chemotherapy.	Complete response rate and safety	Complete response rate 87.7% vs. 67.6% in CCRT arm (*p* = 0.000)
NCT01566240	Induction Chemotherapy Plus Chemoradiation as First Line Treatment for Locally Advanced Cervical Cancer (INTERLACE) [34]	III	500	6 weeks carboplatin AUC2 and paclitaxel 80 mg/m^2^ → CCRT in week 7	PFS and OS	5-year PFS 73% vs. 64% with CCRT alone (HR 0.65); 5-year OS 80% vs. 72% with CCRT alone (HR 0.61)
NCT00193739	Neoadjuvant Chemotherapy Followed by Radical Surgery versus Concomitant Chemotherapy and Radiotherapy in Patients With Stage IB2, IIA, or IIB Squamous Cervical Cancer: A Randomized Controlled Trial [36]	III	635	paclitaxel 175 mg/m^2^+ carboplatin AUC5/6 for 3 cycles q3w → surgery 3–4 weeks after the completion of chemotherapy	DFS	5-year DFS significantly lower in the NACHT–surgery arm than in the CCRT arm (69.3% versus 76.7%; HR = 1.38)
NCT00039338	Chemotherapy Followed by Surgery vs. Radiotherapy Plus Chemotherapy in Patients With Stage IB or II Cervical Cancer (EORTC-55994) [37]	III	626	platin-based chemotherapy (minimum cumulative cisplatin dose 225 mg/m^2^) → surgery within 6 weeks	OS	5-year OS 72% for the NACHT–surgery arm and 76% for the CCRT arm

### 3.3. Adjuvant Chemotherapy

The hypothesis that adjuvant systemic therapy after CCRT could have the potential to reduce the risk of distant metastasis and improve survival led to the emergence of numerous studies; however, only few of them are randomized controlled trials. A recent meta-analysis [38] evaluated the efficacy and safety data of 12 studies on adjuvant treatment using the two most frequently employed chemotherapy doublets (platinum–taxane and platinum–pyrimidine antagonists). No significant improvement in overall survival was observed for either schedule: the pooled HR for overall survival was 0.76 (*p* = 0.22) and 0.47 (*p* = 0.16) for the addition to CCRT of a platinum–pyrimidine antagonist or platinum–taxane, respectively. Completion rates were 82% for platinum–pyrimidine antagonist and 74% for platinum–taxane. Severe hematological and gastro-intestinal toxicities were significantly increased by adding adjuvant chemotherapy to CCRT.

The phase III study performed by Duenas-Gonzales et al. [39] on radiotherapy with concurrent cisplatin versus radiotherapy with concurrent cisplatin–gemcitabine followed by adjuvant cisplatin–gemcitabine chemotherapy was the only randomized controlled trial showing a significant improvement in PFS (3-year PFS 74.4% versus 65%, HR 0.68) and OS (3-year OS with HR 0.68) in the CCRT plus adjuvant chemotherapy arm. High-grade toxicities were more frequent in the experimental arm (86.5% versus 46.3 in the control arm, *p* < 0.001), including two deaths possibly related to treatment toxicity.

The most recent evidence on adjuvant chemotherapy after standard CCRT in locally advanced cervical cancer comes from the multicenter, randomized, phase III OUTBACK trial (NCT01414608) [40]. A total of 926 patients with FIGO 2008 stage IB1 disease with nodal involvement or stage IB2, II, IIIB, or IVA cervical cancer were included and randomly assigned to receive standard cisplatin-based CCRT or standard CCRT followed by adjuvant chemotherapy with four cycles of carboplatin AUC5 and paclitaxel 155 mg/m^2^ every three weeks. The primary endpoint was 5-year overall survival. The secondary endpoints were, among others, 3-year and 5-year PFS and safety. The implementation of adjuvant chemotherapy after CCRT did not improve outcomes compared with CCRT alone: 5-year overall survival was 72% in the adjuvant chemotherapy group versus 71% in the control group (HR 0.9), and a similar pattern was seen for PFS. In addition, it increased short-term toxicities, mainly neutropenia (20% in the adjuvant chemotherapy group versus 8% in the CCRT only group) and anemia (18% versus 8%, respectively).

### 3.4. Immunotherapy

The rationale for the use of immunotherapy in cervical cancer finds its cornerstone in the interactions between the immune system, human papilloma virus (HPV) infection, and cervical cancer progression [41]. Indeed, due to its viral origin, cervical cancer is characterized by a specific immunologic profile and approximately 20% of cases exhibit a high tumor mutational burden, indicating tumor antigenicity [42]. In addition, high expression of immune checkpoints, such as Cytotoxic T lymphocyte antigen 4 (CTLA4) and programmed cell death 1/programmed cell death-ligand 1 (PD1/PD-L1), has been demonstrated in intraepithelial lesions and cervical cancer [43]. Furthermore, cervical cancer reveals its strong immunogenic potential through the presence of stromal tumor-infiltrating lymphocytes (TILs) [44]. These data have provided a robust biological rationale for the numerous studies investigating the role of immunotherapy in cervical cancer treatment.

In the first line setting, the Keynote-826 trial [45] investigated the association of pembrolizumab to chemotherapy, with or without bevacizumab, for 617 patients with persistent, recurrent, or metastatic cervical cancer. The addition of pembrolizumab significantly improved PFS and 2-year OS in patients with PD-L1 expression scores ≥ 1 (PFS 10.4 vs. 8.2 months, HR 0.62, *p* < 0.001; 2-year OS 53% vs. 41.7%, HR 0.64, *p* < 0.001) and for those with PD-L1 expression scores ≥ 10 (PFS 10.4 vs. 8.1 months, HR 0.58, *p* < 0.001; 2-year OS 54.4 vs. 44.6 months, HR 0.61, *p* = 0.001).

In the second-line setting, the Empower-Cervical 1 trial [46] evaluated the efficacy of cemiplimab versus investigator’s choice single-agent chemotherapy in patients who had disease progression after first-line platinum-based chemotherapy, regardless of their PD-L1 status, demonstrating a benefit in terms of OS (12 vs. 8.5 months, HR 0.69, *p* < 0.001) and PFS (HR 0.75, *p* < 0.001).

New evidence is emerging about the role of immune checkpoint inhibitors in treating locally advanced cervical cancer. The trials examined in this review are summarized in Table 3.

#### 3.4.1. Immune Checkpoint Inhibitors in Association with Chemoradiation Therapy

Several clinical trials investigated the role of immune checkpoint inhibitors in association with chemoradiation in locally advanced cervical cancer.

The randomized, phase III CALLA trial (NCT03830866) [47] evaluated the efficacy of durvalumab, a monoclonal antibody that selectively binds PD-L1, in combination and following standard of care chemoradiotherapy. A total of 1040 patients with FIGO 2009 stage IB2–IIB lymph node-positive or FIGO 2009 stages IIIA–IVA disease were enrolled and randomized to receive either intravenous durvalumab 1500 mg or placebo every four weeks, concurrent with and following standard chemoradiotherapy, for a total of 24 cycles. Progression-free survival was the primary endpoint, while OS was the key secondary endpoint. The addition of durvalumab to standard treatment did not significantly improve progression-free survival: 12-month and 24-month PFS were, respectively, 76.0% and 65.9% with durvalumab versus 73.3% and 62.1% with placebo. In post hoc analysis, a favorable HR for PFS was identified for durvalumab versus placebo in patients with a PD-L1 TAP (tumor area positivity) score 20% or greater, regardless of lymph node involvement. The HR for overall survival with durvalumab versus placebo was 0.78.

The safety of pembrolizumab in combination with CCRT was assessed by an open-label randomized phase II study (NCT02635360) [48]. Eighty-eight patients with locally advanced cervical cancer were included and administered pembrolizumab sequentially or during standard CCRT. The study results supported the safety and feasibility of adding pembrolizumab to pelvic CCRT concurrently or sequentially, since no significant difference was observed in the two arms.

The phase III, randomized, placebo-controlled ENGOT-cx11/KEYNOTE-A18 study (NCT04221945) [49] investigated the efficacy and safety of pembrolizumab combined with CCRT in high-risk locally advanced cervical cancer (FIGO 2014 [50] stage IB2–IIB node-positive or stage III–IVA disease). A total of 1060 patients were randomized to receive pembrolizumab or placebo together with CCRT followed by 15 cycles of pembrolizumab or placebo every 6 weeks. The primary endpoints were progression-free survival (PFS) as assessed by the investigator or by confirming suspected disease progression through histopathologic examination and overall survival (OS). The first interim analysis, at a median follow-up of 17.9 months, showed a statistically significant improvement in PFS (24-month PFS was 67.8% with pembrolizumab + CCRT versus 57.3% with placebo + CCRT) and a favorable trend in OS (HR = 0.73) for the combination pembrolizumab and CCRT compared with placebo and CCRT, with a manageable safety profile. In March 2024, a Merk press release announced that the addition of pembrolizumab to CCRT resulted in a statistically significant and clinically meaningful improvement in OS [51].

The efficacy of atezolizumab concurrently and after CCRT is being assessed by the randomized, open-label, phase II ATEZOLACC trial (NCT03612791). The study is currently recruiting high-risk patients, in particular FIGO 2009 Stages IB1–IIA with positive pelvic lymph nodes, stages IIB–IVA, and any stage with positive para-aortic lymph nodes. Its primary endpoint is PFS. Study completion is estimated in July 2024.

The phase I NiCOL trial (NCT03298893) [52] explored the anti-PD-1 nivolumab concurrent and following CCRT in 21 patients with stages IB2 to IVA locally advanced cervical cancer, regardless of lymph node status. The primary endpoint was the safety and tolerability profile; secondary endpoints included objective response rate, progression-free survival, disease-free survival, and immune correlates of response. The study reported the safety and tolerability of concomitant nivolumab plus definitive CCRT: three dose-limiting toxicities were observed, corresponding to hypotension and acute kidney failure, which were related to cisplatin administration. Initial oncologic outcomes were promising, with an overall response rate of 93.8% and 2-year PFS of 75%.

#### 3.4.2. Immune Checkpoint Inhibitors Prior to Chemoradiation Therapy

Some studies are questioning the ideal sequencing of immunotherapy and radiation therapy in locally advanced cervical cancer, investigating the role of immune checkpoint inhibitors as primer for chemoradiotherapy.

A phase I randomized trial by J. Mayadev et al. (NCT03738228) [53] recruited FIGO 2009 clinical stage IB3/IIA patients with positive para-aortic nodes and clinical stage IIB/IIIB/IVA patients with positive pelvic or para-aortic lymph nodes, randomized to receive atezolizumab prior to and during CCRT or exclusively during cisplatin chemotherapy and radiation therapy. The primary objective was to determine whether differences in the sequencing of atezolizumab and CCRT resulted in differential immune activation, expressed as clonal expansion of T cell receptor beta repertoires in peripheral blood. The secondary objectives were the feasibility and safety of atezolizumab prior to and concurrent with chemoradiation and the correlation of T cell receptor profile and PD-L1 expression with post-treatment PET-CT scan and 2 years’ disease-free survival. The study results have not yet been published.

The phase II COLIBRI trial (NCT04256213) [54] evaluated the biological impact of nivolumab + ipilimumab followed by standard chemoradiation therapy in locally advanced cervical cancer. The aim of the study was to evaluate the evolution of the CD8+/FOXP3+ ratio of lymphocytes before and after nivolumab + ipilimumab administration through multiplex-immunofluorescence prior to CCRT. Additionally, gene expression profiling was used to assess the 27-gene based “HOT” score associated with immunologically active tumors. An increase in total CD8+ cells, proliferating CD8+ cells, and the CD8+/FOXP3+ ratio was observed between baseline and the start of CCRT. A significant increase in the expression of the CD8A gene and the ‘HOT’ score was observed after nivolumab + ipilimumab administration. The complete response rate at the end of the treatment was 82.5% and partial responses were recorded in 40% of patients. Global response was 81% in patients with FIGO I/II and 74% in patients with FIGO III/IV stage. The clinical endpoints of PFS and OS are expected in 2025.

#### 3.4.3. Immune-Based Neoadjuvant Treatment

The single-arm, phase II NACI study (NCT04516616) [55] investigated the activity and safety of the PD1 inhibitor camrelizumab combined with neoadjuvant chemotherapy followed by radical hysterectomy in patients with PD-L1-positive locally advanced cervical cancer at FIGO 2018 stage IB3, IIA2, or IIB/IIIC1 with a tumor diameter ≥ 4 cm. Patients received one cycle of nab-paclitaxel and cisplatin followed by two cycles of camrelizumab combined with chemotherapy. The primary endpoint was the objective response rate. The secondary endpoints were, among others, the rate of pathological complete response and safety. For the 85 patients enrolled, the objective response rate was 98%, and a pathological complete response was observed in 38% of patients submitted to surgery. No serious adverse events or treatment-related deaths occurred.

Two other studies investigating immune-based neoadjuvant therapy are ongoing. One of these trials (NCT04799639) [56] enrolled stage IB3 and IIA2 cervical cancer patients receiving neoadjuvant chemotherapy with paclitaxel and cisplatin plus sintilimab for three cycles followed by radical surgery. The primary endpoint of pathological complete response rate was 35%; the objective response rate was 95%. Study completion is estimated in March 2026. The second one is the MITO CERV 3 trial (NCT04238988), investigating the role of pembrolizumab in combination with carboplatin–paclitaxel. The study includes patients with locally advanced cervical cancer (stages IB2–IIB) with a Combined Positive Score (CPS) ≥ 1. After three cycles of neo-adjuvant platinum-based chemotherapy associated with pembrolizumab, non-progressing patients undergo radical surgery. After surgery, patients presenting high-risk factors receive three cycles of adjuvant carboplatin–paclitaxel chemotherapy plus pembrolizumab followed by maintenance with pembrolizumab. The primary objective is 2-year PFS. Secondary objectives include OS, clinical response rate, pathologic optimal response and safety.

#### 3.4.4. Maintenance Treatment

The phase II randomized ATOMICC trial (NCT03833479) [57] was designed to evaluate the role of the anti-PD1 dostarlimab as maintenance therapy in high-risk locally advanced cervical cancer (FIGO 2009 stages IB2, IIA2, and IIB with at least two positive pelvic nodes; stages IIIA, IIIB, IVA; or any stage with at least one positive para-aortic lymph node) who achieved a partial or complete response after CCRT. The primary endpoint is progression-free survival. The secondary objectives include overall survival, toxicity assessment, and health-related quality of life. Results are expected to be released in December 2025.

The randomized phase III e-VOLVECervical trial (NCT06079671) is recruiting FIGO 2018 Stage IIIC to IVA cervical cancer patients in order to evaluate the efficacy of the monovalent bispecific human IgG1 monoclonal antibody volrustomig as maintenance treatment. PFS in participants with PD-L1 expression is the primary endpoint. Secondary endpoints include PFS regardless of PD-L1 expression and OS in participants with PD-L1 expression and regardless of PD-L1 expression. Study completion is estimated in October 2029.

#### 3.4.5. Emerging Immunotherapy Strategies

While checkpoint inhibitors represent the most extensively studied immunotherapy for cervical cancer, numerous alternative approaches to stimulate the immune system have been proposed. Among them, therapeutic vaccines and cell-based therapy are the most promising strategies, with encouraging results in many pre-clinical trials [58].

The integration of preventative HPV vaccines into clinical practice heralded a new era in the landscape of HPV-related disease. They prevent HPV infections by stimulating the generation of neutralizing antibodies that bind viral particles, hindering their entry into host cells. Moreover, the well-known pathogenic implication of HPV in cervical cancer promoted several investigations on therapeutic vaccines in the preinvasive and invasive disease treatment. The HPV oncogenes E6 and E7, involved in the pathological mechanism of cellular transformation, are the targets of the majority of the tested therapeutic vaccines for cervical cancer.

Although therapeutic vaccines and cell-based therapies are primarily being investigated in metastatic and recurrent settings, some studies are exploring their role in locally advanced cervical cancer. Moreover, these treatments may synergistically enhance immune response when combined with radiation therapy, optimizing treatment efficacy.

Based on previous encouraging data from two phase II studies on metastatic and recurrent disease [59,60], the phase III AIM2CERV clinical trial was designed to assess the effectiveness of axalimogene filolisbac (AXAL) after the completion of CCRT in high-risk locally advanced cervical cancer (FIGO stage I–II with positive pelvic nodes, stage III–IVA, and any stage with para-aortic nodes). Axalimogene filolisbac is a live attenuated Listeria monocytogenes vector system which infects host antigen-presenting cells and secretes a fusion HPV E7 protein [61]. Unfortunately, the study was closed in 2019 before full accrual was reached and no results have been published yet.

The ongoing single-arm, phase II IMMUNOCERV trial (NCT04580771) [62] is currently assessing a liposomal HPV-16 E6/E7 T-cell activating immunotherapy (PDS0101) combined with CCRT in advanced cervical cancer patients with either lymph node metastasis or tumors of >5 cm. An interim analysis conducted on eight patients that completed the treatment showed a complete response rate of 87.5% on PET at 3 months; the 1-year disease-free survival rate was 85.7%, and the 1-year overall survival 100%, with a favorable toxicity profile.

**Table 3:** Immune checkpoint inhibitors in locally advanced cervical cancer.

Clinical Trial Registry Identifier	Title	Phase	Patients	Intervention	Primary Endpoint (s)	Outcome (s)
NCT03830866	Durvalumab With Chemoradiotherapy for Women With Locally Advanced Cervical Cancer (CALLA) [47]	III	770	Concurrently and after CCRT	PFS	12 m and 24 m PFS 76.0% and 65.9% with durvalumab vs. 73.3% and 62.1% with placebo
NCT02635360	Pembrolizumab and Chemoradiation Treatment for Advanced Cervical Cancer [48]	II	88	Concurrently and after CCRT	Safety and feasibility	No significant difference in the two arms
NCT04221945	Pembrolizumab plus chemoradiotherapy for high-risk locally advanced cervical cancer (ENGOT-cx11/KEYNOTE-A18) [49]	III	1060	Concurrently CCRT	PFS and OS	24 m PFS 67.8% with pembrolizumab + CCRT vs. 57.3% with placebo + CCRT. Significant benefit in OS
NCT03612791	Trial Assessing the Inhibitor of Programmed Cell Death Ligand 1 (PD-L1) Immune Checkpoint Atezolizumab in locally advanced cervical cancer (ATEZOLACC)	II	189	Concurrently and after CCRT	PFS	Study completion estimated in July 2024
NCT03298893	Nivolumab in Association With Radiotherapy and Cisplatin in Locally Advanced Cervical Cancers Followed by Adjuvant Nivolumab for up to 6 Months (NiCOL) [52]	I	21	Concurrently and after CCRT	Safety and tolerability	Favorable safety profile
NCT03738228	Atezolizumab as an Immune Primer and Concurrently with CCRT for Node Positive Locally Advanced Cervical Cancer [53]	I	40	Before and concurrently CCRT	Impact of differences in sequencing of atezolizumab and CCRT on immune activation	Study completion estimated in September 2024
NCT04256213	In situ immune impact of neoadjuvant nivolumab + ipilimumab before standard chemoradiation therapy for FIGO Ib3-IVa squamous cervical carcinoma patients (COLIBRI) [54]	II	40	Before CCRT	Evolution of the CD8+/FOXP3+ lymphocyte ratio before and after nivolumab + ipilimumab combination	Increase in total CD8+ cells, proliferating CD8+ cells, and the CD8+/FOXP3+ ratio between baseline and the start of CCRT
NCT04516616	Neoadjuvant camrelizumab plus chemotherapy for locally advanced cervical cancer (NACI) [55]	II	85	Combined with NACHT	Pathological complete response rate	98%
NCT04799639	Efficacy and safety of sintilimab plus paclitaxel and cisplatin as neoadjuvant therapy for locally advanced cervical cancer [56]	II	47 (estimated)	Combined with NACHT	Pathological complete response rate	35% (study completion estimated in March 2026)
NCT04238988	Carboplatin-Paclitaxel-Pembrolizumab in Neoadjuvant Treatment of Locally Advanced Cervical Cancer (MITO CERV 3)	II	45 (estimated)	Combined with NACHT	PFS	Pending
NCT03833479	Dostarlimab as maintenance therapy for patients with high-risk locally advanced cervical cancer after chemoradiation (ATOMICC) [57]	II	132 (estimated)	Maintenance therapy	PFS	Study completion estimated in December 2025
NCT06079671	Study of Volrustomig in Women with High Risk Locally Advanced Cervical Cancer (e-VOLVECervical)	III	1000 (estimated)	Maintenance therapy	PFS	Study completion estimated in October 2029

## 4. Discussion

Despite the implementation of targeted vaccines and screening protocols, cervical carcinoma continues to be one of the most common malignancies in women worldwide, demonstrating an unfavorable prognosis upon advanced-stage detection. The presence of para-aortic lymph node metastasis is the most important prognostic factor, predicting a higher risk of distant recurrence [63,64]; in addition, it guides radiation field planning in order to optimize disease control and, at the same time, avoid overtreatment and unnecessary toxicity. In this context, the benefit of a surgical para-aortic staging is still an open question. The first randomized trial addressing this issue, published in 2003 by Lai C-H et al., had a limited sample size and was prematurely stopped after an interim analysis showed a significant reduction in the survival of surgically staged patients [65]. More recently, the Uterus-11 study [15] did not reveal a benefit in terms of DFS and OS for patients undergoing surgical staging; however, this experience demonstrated that surgical staging is feasible, without significantly delaying the initiation of definitive concurrent chemoradiotherapy or increasing radiation toxicity [66]. The results of the PAROLA trial [16] are anticipated to clarify the impact of surgical nodal staging on survival in PET-CT FIGO stage IIIC1 cervical cancer patients.

Regarding radiation therapy and brachytherapy, the notable progress achieved in refining techniques, such as the implementation of Intensity-Modulated Radiation Therapy/Volumetric Modulated Arc Therapy (IMRT/VMAT) and image-guided adaptive brachytherapy (IGABT) have enabled more targeted and effective tumor eradication with a lower incidence of grade 3 toxicities. The EMBRACE II study [24] is applying a high-quality treatment protocol using these technologies to achieve a high level of local, nodal, and systemic control while minimizing morbidity. Notably, the recommendations tested in the EMBRACE II study have been incorporated into the ESGO-ESTRO-ESP Guidelines [6].

Although the results from previous trials on neoadjuvant chemotherapy before CCRT are controversial, the Interlace study [34] demonstrated that induction chemotherapy is a feasible, cost-effective, and well-tolerated approach that brings a significant benefit in terms of survival. In addition, induction chemotherapy drugs are widely available and more affordable compared to immunotherapy, eliminating potential economic barriers to adopting this treatment schedule. A large proportion of the recruited patients had node-negative disease and 77% of patients were stage II, suggesting that this strategy could be more suitable for a low-risk population. The precise timing of seven days between the end of induction chemotherapy and the beginning of CCRT applied in the study may have had a significant impact on the outcomes.

Two large phase III randomized trial failed to demonstrate a survival benefit of NACHT followed by surgery compared to standard CCRT in locally advanced cervical cancer [36,37]. Specifically, Gupta et al. found a significant difference in favor of CCRT in terms of disease-free survival (*p* = 0.038), but no difference in overall survival. Similarly, the most recent EORTC-55994 study reported no difference in OS between the two arms, while PFS was marginally better for the CCRT arm. Based on the results of these studies and on current guidelines, neoadjuvant chemotherapy followed by surgery cannot be considered a standard of care in patients with locally advanced cervical cancer. However, the acceptable morbidity and lower prevalence of vaginal and long-term toxicities associated with NACHT followed by surgery still make this strategy appealing for a young and carefully selected population. Adequately powered prospective studies are needed to address this issue [67]. Additionally, in middle- and low-income countries with limited access to radiotherapy facilities, NACHT followed by radical surgery can be an acceptable alternative to CCRT.

Solid data supporting the integration of adjuvant chemotherapy in LACC treatment are still lacking. The only randomized controlled trial that demonstrated a survival benefit [39] had a key difference in the initial CCRT treatment between its two arms: the CCRT arm received six cycles of cisplatin, while the experimental arm received six cycles of concurrent cisplatin and gemcitabine. This discrepancy made it impossible to draw definitive conclusions from the study. Furthermore, the experimental arm experienced a significantly higher incidence of severe toxicities. The recently published OUTBACK trial [40], which administered cisplatin concurrently with radiotherapy in both arms followed by four courses of a carboplatin–paclitaxel in the experimental arm, failed to demonstrate a benefit from adding adjuvant treatment after CCRT. A limitation of the trial could be the low initiation and completion rates of adjuvant chemotherapy [68].

Immunotherapy is a promising field in cervical cancer treatment and is currently being assessed in various settings and providing interesting findings. Although the CALLA study [47] did not demonstrate a survival benefit for the combination of durvalumab with CCRT, the phase III ENGOT-cx11/KEYNOTE-A18 trial [49] revealed a benefit in terms of PFS and OS for the addition of concurrent and maintenance pembrolizumab to chemoradiotherapy. The disparity in outcomes between these two studies could be due to the different agents investigated, with a possible superiority of PD-1 inhibitors over PD-L1 inhibitors. Furthermore, when comparing the PFS curves of the two studies, it is evident that the 2-year PFS for the control group in the CALLA study (62.1%) was higher than the 2-year PFS of the control group in the KEYNOTE A18 study (57.3%). This discrepancy might have led to an underestimation of the impact of adding immunotherapy in the CALLA study. The promising results of the KEYNOTE-A18 trial may potentially change the standard of care for high-risk locally advanced cervical cancer patients.

## 5. Conclusions

Locally advanced cervical cancer is a heterogeneous entity, and its management faces numerous challenges. This scenario underscores the demand for the exploration of new therapeutic modalities; therefore, new evidence has recently emerged in different treatment fields. In this context, patient selection for the most suitable treatment approach according to risk stratification, age, and potential benefits from immunotherapy remains a crucial aspect.
